# Development and external validation of an interpretable machine learning model for early prediction of in-hospital mortality in neurocritical care

**DOI:** 10.3389/fneur.2026.1888888

**Published:** 2026-09-24

**Authors:** Xiao Cheng, Jianxin Ye, Hongen Wei

**Affiliations:** 1 The Neurology Department of Shanxi Provincial People's Hospital, Shanxi Medical University, Shanxi Key Laboratory of Neurobehavior and Brain Disease Control, Taiyuan, China; 2 Department of Neurology, The 900th Hospital of the Joint Logistics Support Force of the Chinese People's Liberation Army, Fuzhou, China

**Keywords:** clinical decision support, eICU-CRD, external validation, in-hospital mortality, machine learning, MIMIC-IV, neurocritical care, SHAP

## Abstract

**Background:**

Early risk assessment is a routine part of neurocritical care, yet mortality prediction remains difficult because neurological injury often coexists with respiratory, circulatory, renal, and inflammatory dysfunction. This study aimed to develop and externally validate an interpretable machine learning (ML) model for predicting in-hospital mortality in neurocritical-care patients using variables available within the first 24 h after intensive care unit (ICU) admission.

**Methods:**

We conducted a retrospective cohort study using two public critical-care databases. The Medical Information Mart for Intensive Care IV (MIMIC-IV) database was used for model development and internal testing, and the eICU Collaborative Research Database (eICU-CRD) was used for independent external validation. The report follows the Transparent Reporting of a Multivariable Prediction Model for Individual Prognosis or Diagnosis (TRIPOD) statement. Adult ICU patients with neurocritical-care diagnoses were identified by predefined International Classification of Diseases codes. Candidate predictors were restricted to information recorded during the first 24 h after ICU admission and included demographics, comorbidities, vital signs, laboratory results, neurological status, organ-support therapies, and severity scores. Least absolute shrinkage and selection operator (LASSO) regression was used for feature selection. Logistic regression, random forest, XGBoost, LightGBM, CatBoost, and multilayer perceptron models were trained. Discrimination was assessed by area under the receiver operating characteristic curve (AUROC) and area under the precision-recall curve (AUPRC). Calibration, decision curve analysis (DCA), and SHapley Additive exPlanations (SHAP) were used to evaluate probability reliability, potential clinical utility, and interpretability. The operating threshold used for classification metrics was fixed in the MIMIC-IV development data before internal and external evaluation.

**Results:**

The MIMIC-IV cohort included **6,842** patients, of whom **1,014** died during hospitalization; the held-out internal test set contained **1,368** patients and **203** deaths. The external validation cohort included **5,236** eICU-CRD patients, of whom **686** died. LightGBM showed the best overall performance. In the internal test cohort, it achieved an AUROC of **0.841** (95% CI: **0.821–0.861**) and an AUPRC of **0.518** (95% CI: **0.486–0.552**). In external validation, the AUROC was **0.801** (95% CI: **0.779–0.824**) and the AUPRC was **0.413** (95% CI: **0.380–0.449**), with sensitivity of **72.8%**, specificity of **73.9%**, positive predictive value of **30.3%**, negative predictive value of **94.7%**, and F1 score of **0.428**. Thus, approximately 70% of patients classified as high risk at this operating point survived to hospital discharge. Calibration was acceptable in the external cohort, with a Brier score of **0.101**, calibration intercept of **0.050**, and calibration slope of **0.84**. SHAP analysis identified GCS total score, invasive mechanical ventilation, age, lactate, SOFA score, vasopressor use, and creatinine as leading contributors to predicted mortality risk.

**Conclusions:**

A model based on routinely collected first-day ICU data provided externally validated and interpretable risk stratification for in-hospital mortality in neurocritical care. Its low positive predictive value at the reported operating point precludes use as a stand-alone basis for prognostic pessimism, treatment limitation, or withdrawal of life-sustaining treatment. Prospective validation in diverse countries and health systems is required before clinical implementation.

## 1 Introduction

Neurocritical care includes patients with acute neurological injury who require intensive monitoring and organ support. These patients may present with ischemic stroke, intracerebral hemorrhage, subarachnoid hemorrhage, traumatic brain injury, status epilepticus, central nervous system infection, or other severe neurological disorders. The burden of neurological disease remains high worldwide, and many affected patients require ICU-level care during the acute phase (1–3). In this setting, early prediction of in-hospital mortality may support structured reassessment and monitoring, but any prognostic estimate must be interpreted in conjunction with the clinical trajectory, patient preferences, and the uncertainty inherent in early neuroprognostication (4–8).

A specific concern in neurocritical-care prediction is self-fulfilling prophecy bias. Death may follow decisions to withhold or withdraw life-sustaining treatment (WLST), and those decisions can themselves be influenced by depressed consciousness, neurological severity, age, and early organ-support requirements. A model trained on observed in-hospital mortality may therefore learn elements of historical treatment-limitation practice in addition to biological risk. This issue is central to the interpretation and safe use of mortality predictions.

Prognosis after acute neurological injury is not determined by brain injury alone. Impaired consciousness, respiratory failure, circulatory instability, renal dysfunction, infection, coagulopathy, and systemic inflammation often occur together during the first ICU day. These extracranial complications may worsen cerebral perfusion, limit treatment options, and increase mortality risk. A useful early prediction model should therefore combine neurological status with systemic physiology rather than rely only on diagnostic labels.

Conventional scores remain important in critical care. The Glasgow Coma Scale (GCS), Sequential Organ Failure Assessment (SOFA), and Acute Physiology and Chronic Health Evaluation II (APACHE II) are widely used because they are transparent and familiar to clinicians (9–11). However, these scores use predefined variables and largely additive structures. They may not fully capture nonlinear relationships among neurological injury, laboratory abnormalities, treatment intensity, and organ dysfunction. Previous work has also shown that clinician judgement and conventional scoring systems may vary in accuracy when used for ICU mortality prediction (12, 13).

Machine learning can model complex interactions in electronic health record data and has been applied to acute stroke, traumatic brain injury, subarachnoid hemorrhage, sepsis, acute kidney injury, and ICU mortality prediction (14–22). Dynamic and real-time ICU models have further shown the value of using high-frequency clinical data for mortality prediction (23–26). Nevertheless, many medical AI studies remain limited by single-database development, unclear prediction timing, incomplete calibration assessment, and limited evaluation of clinical utility (27–32).

The first 24 h after ICU admission provide a practical prediction window. By that time, early neurological severity, vital signs, laboratory results, and initial organ-support requirements are usually available. At the same time, the window remains early enough to support clinical action. Restricting predictors to this period also reduces information leakage from later complications, treatment escalation, or outcome-proximal events.

In this study, we developed and externally validated an interpretable ML model for prediction of in-hospital mortality at a fixed 24-h ICU landmark in a heterogeneous neurocritical-care cohort. MIMIC-IV was used for development and internal testing, and eICU-CRD was used for independent external validation. Candidate predictors were restricted to the first 24 h after ICU admission. The final model was evaluated for discrimination, calibration, decision-curve utility, subgroup stability, and SHAP-based interpretability. The report follows TRIPOD, and the intended role of the model is risk stratification rather than autonomous treatment or WLST decision making.

## 2 Materials and methods

### 2.1 Study design and reporting framework

This retrospective prediction-modeling study used routinely collected ICU data to estimate the risk of in-hospital mortality among neurocritical-care patients. MIMIC-IV was used for model development and internal validation, and eICU-CRD was used for external validation. The manuscript was prepared in accordance with the TRIPOD statement, with complementary consideration of STROBE, RECORD, and updated TRIPOD+AI guidance (29, 31, 33, 34). A completed 22-item TRIPOD checklist is provided in Supplementary Table S1. To avoid information leakage, all predictors were restricted to the first 24 h after ICU admission. The cohort construction and model-development workflow is summarized in Figure 1. The fixed 24-h prediction interface and downstream evaluation pipeline are detailed in Figure 2.

**Figure 1:**
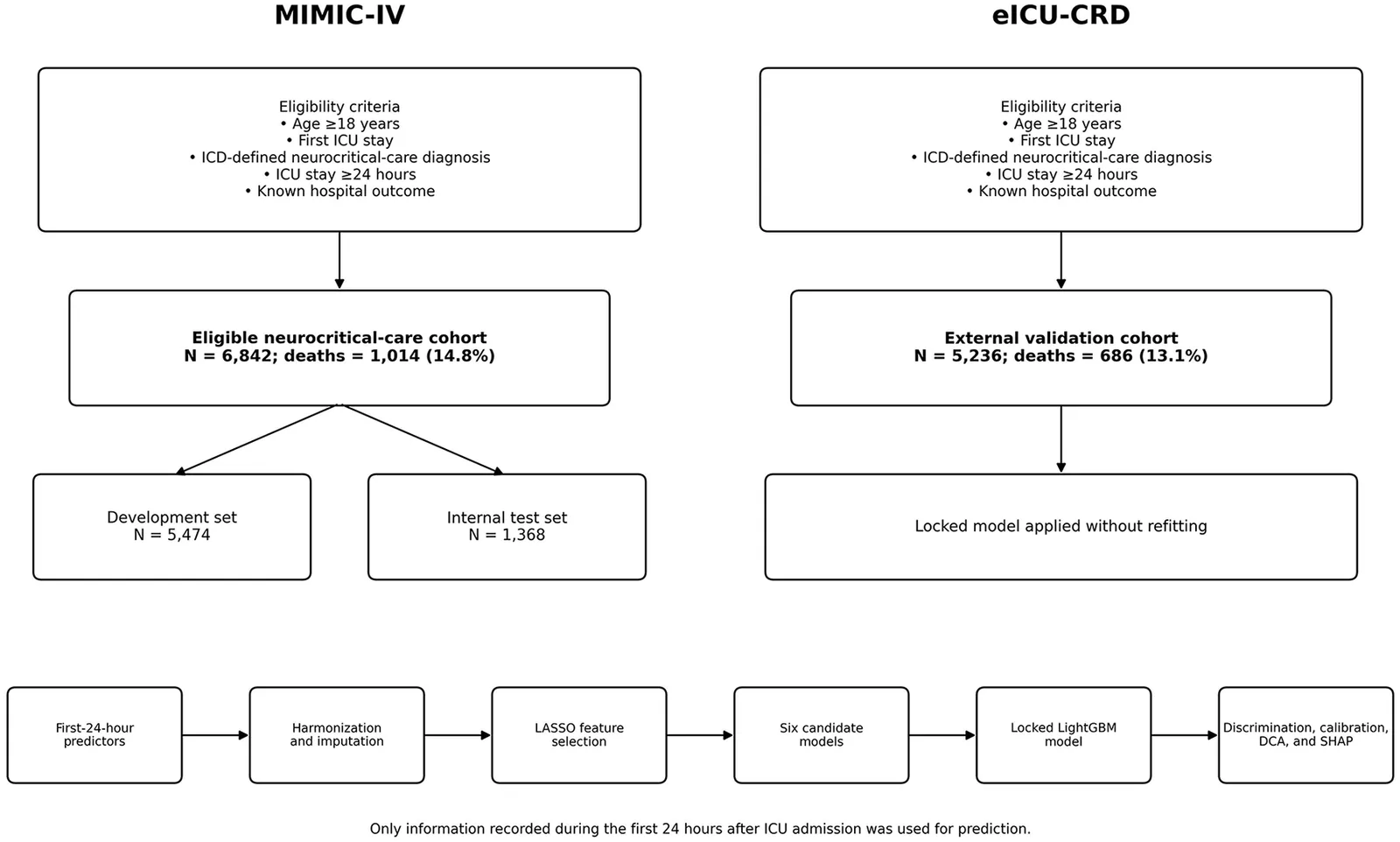
Study design and model-development workflow. MIMIC-IV was used for model development and internal testing, whereas eICU-CRD served as the external validation cohort. Eligibility required age 18 years or older, a first ICU stay, an ICD-defined neurocritical-care diagnosis, an ICU stay of at least 24 h, and known hospital outcome status. Only variables recorded during the first 24 h were used for prediction.

**Figure 2:**
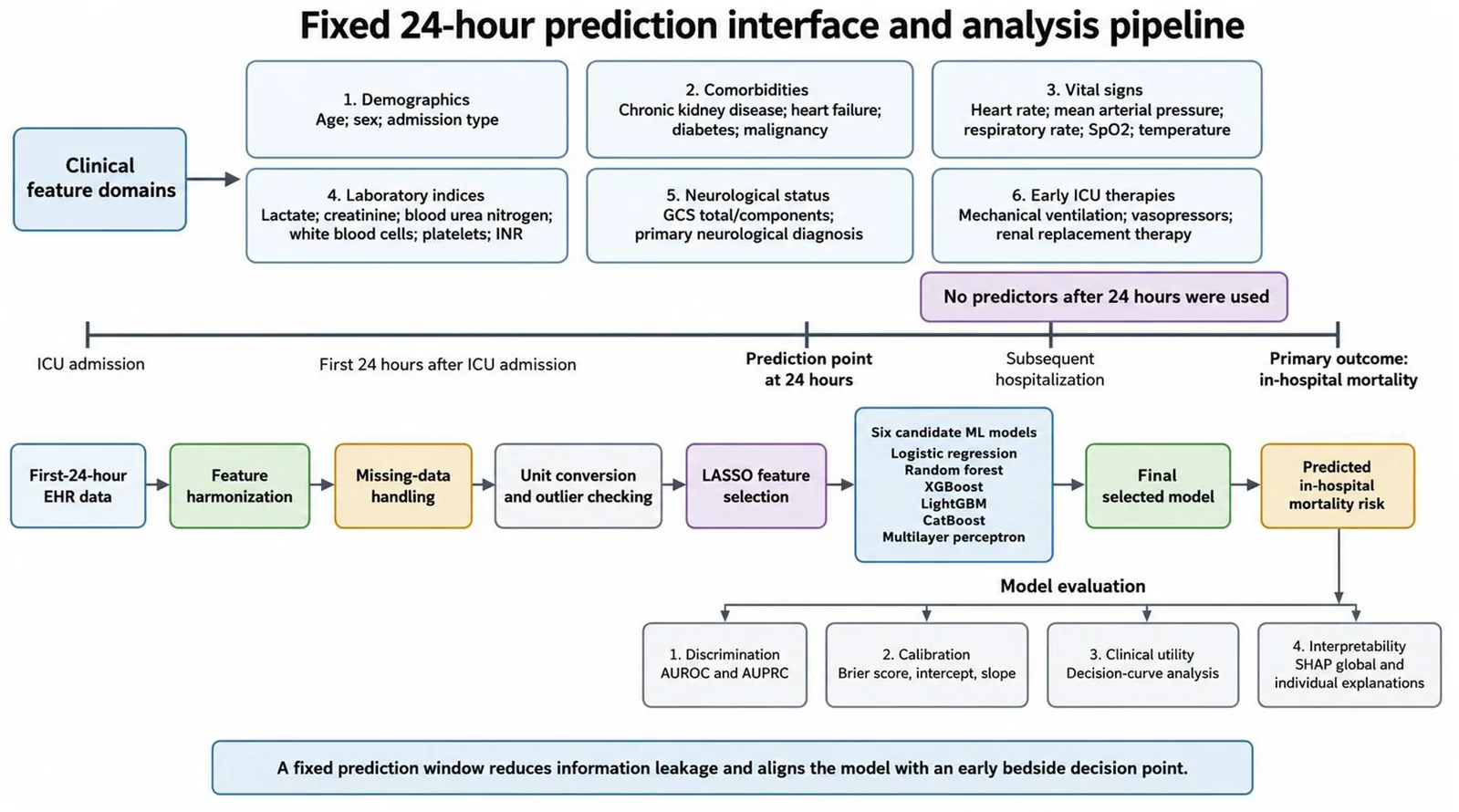
Fixed 24-h prediction interface and analysis pipeline. Clinical variables were extracted only from the first 24 h after ICU admission and grouped into demographics, comorbidities, vital signs, laboratory indices, neurological status, and early ICU therapies. After feature harmonization, missing-data handling, unit conversion, outlier checking, and LASSO feature selection, six candidate machine-learning models were trained. The final model was evaluated for discrimination, calibration, clinical utility, and interpretability while avoiding use of information recorded after the prediction time point.

### 2.2 Data sources

MIMIC-IV contains deidentified electronic health record data from ICU admissions at Beth Israel Deaconess Medical Center (35). It includes demographics, diagnoses, laboratory tests, vital signs, procedures, severity scores, treatments, and outcomes. eICU-CRD is a multicenter critical-care database derived from hospitals across the United States and includes high-granularity physiological, laboratory, treatment, and outcome data (36). The databases also reflect different institutional practices, including thresholds for intubation, vasopressor initiation, laboratory testing, and transfer to intensive care. These differences were treated as clinically relevant sources of transportability challenge rather than as evidence that identical care processes existed across cohorts.

### 2.3 Study population

Adult ICU patients were eligible if they were aged 18 years or older and had at least one neurocritical-care diagnosis during the index hospitalization. Diagnoses were identified using predefined ICD-9 and ICD-10 codes recorded in the source databases. The diagnostic groups included ischemic stroke, intracerebral hemorrhage, subarachnoid hemorrhage, traumatic brain injury, status epilepticus, central nervous system infection, and other severe neurological disorders requiring ICU care. Because the diagnosis codes were not independently adjudicated, differences in coding completeness, principal-diagnosis assignment, and institutional billing practice were recognized as potential sources of selection and misclassification bias.

For patients with more than one ICU admission during the same hospitalization, only the first ICU stay was retained. Patients were excluded if they were younger than 18 years, had an ICU stay shorter than 24 h, lacked essential time-stamped information during the first 24 h, or had missing hospital-outcome status. The 24-h minimum ICU stay was required because the model was designed to make predictions at the end of the first ICU day.

### 2.4 Outcome definition

The primary outcome was in-hospital mortality, defined as death during the index hospitalization after ICU admission. Patients discharged alive were classified as survivors. The endpoint therefore represented observed mortality under the care delivered in each database. WLST and comfort-care transitions were not captured with sufficiently consistent definitions in both databases to distinguish deaths following treatment limitation from deaths despite continued life-sustaining therapy. Secondary analyses considered ICU mortality, prolonged ICU length of stay, prolonged invasive mechanical ventilation, and a composite adverse outcome, but the main analysis focused on in-hospital mortality.

### 2.5 Fixed 24-h prediction interface

Candidate predictors were extracted from the first 24 h after ICU admission. For repeatedly measured variables, summary values were calculated using pre-specified rules according to the clinical meaning of each variable, including first, minimum, maximum, or mean values. Treatment variables were encoded as binary indicators when a therapy or organ-support intervention was used during the first ICU day. These variables describe clinical status and treatment decisions before the prediction time point; they should not be interpreted as untreated baseline characteristics or causal determinants of mortality.

Predictors were grouped into demographics, comorbidities, vital signs, laboratory indices, neurological status, and early ICU therapies. The interface included age, sex, admission characteristics, chronic diseases, heart rate, blood pressure, respiratory rate, temperature, oxygen saturation, blood cell counts, renal markers, electrolytes, coagulation tests, glucose, lactate, inflammatory markers when available, GCS components, mechanical ventilation, vasopressor use, renal replacement therapy, and other early organ-support variables. Variables recorded after the 24-h prediction point were not used. The fixed-window design is illustrated in Figure 2, which shows the clinical feature domains, preprocessing sequence, candidate models, and evaluation outputs.

### 2.6 Data preprocessing and feature harmonization

Variables were mapped between MIMIC-IV and eICU-CRD so that each predictor represented the same clinical concept in both databases. Units were converted when necessary. Implausible values were identified using pre-specified clinical ranges and treated as missing; extreme but plausible values were retained. Missingness was reviewed separately by database and survival status before imputation because the decision to obtain a measurement may itself carry prognostic information. Variables with excessive missingness under the pre-specified analysis plan were excluded. For retained variables, multivariate imputation by chained equations was fitted using the MIMIC-IV training data only and then applied without refitting to the internal test and external validation cohorts (37). Categorical variables were encoded as clinically meaningful categories or binary indicators, and continuous variables were standardized when required by the algorithm. A complete-case analysis was not designated as the primary sensitivity analysis because requiring every measurement can preferentially retain patients who underwent more intensive testing and thereby introduce selection bias; robustness was instead examined across alternative missingness thresholds and feature-selection settings.

LASSO regression was used for feature selection in the development cohort (38). Feature selection was performed within the training data only. The selected features were then used to train all candidate models. Variable definitions, missingness summaries, harmonization rules, and feature-selection results are intended for the Supplementary material.

### 2.7 Model development

The MIMIC-IV cohort was split into training and internal test sets using stratified sampling to preserve the in-hospital mortality rate. The training set was used for feature selection, hyperparameter tuning, and model fitting. The internal test set was held out for evaluation within the development database.

Six algorithms were compared: logistic regression, random forest, XGBoost, LightGBM, CatBoost, and multilayer perceptron (39–44). Logistic regression served as an interpretable baseline. Random forest and gradient-boosting methods were included because they can model nonlinear effects and interactions in tabular clinical data. The multilayer perceptron was used as a neural-network comparator.

Hyperparameters were tuned within the training set by cross-validation. Class imbalance was addressed through class weighting and threshold-independent evaluation. The operating threshold used to summarize sensitivity, specificity, predictive values, and F1 score was fixed from the MIMIC-IV development data before evaluation to provide a balanced descriptive operating point; it was applied unchanged in the internal test and eICU-CRD cohorts. This operating point was not intended to function as a treatment-limitation or WLST threshold.

### 2.8 Internal validation and external validation

Internal validation was performed in the held-out MIMIC-IV test set. External validation was then performed in eICU-CRD using the same predictor definitions and preprocessing rules wherever possible. No refitting was performed in the external validation cohort. Performance degradation from internal to external validation was examined as evidence of model transportability.

### 2.9 Model performance evaluation

Discrimination was assessed using AUROC and AUPRC. AUROC measured ranking performance across thresholds. AUPRC was also reported because mortality was less frequent than survival and precision-recall analysis is informative in imbalanced binary classification (45, 46). Sensitivity, specificity, positive predictive value, negative predictive value, accuracy, and F1 score were calculated at the pre-specified operating threshold. Because predictive values depend on outcome prevalence, PPV and the associated false-positive burden were interpreted explicitly in each cohort.

Calibration was assessed using calibration plots, Brier score, calibration intercept, and calibration slope (47, 48). DCA was used to estimate net benefit across clinically plausible threshold probabilities (49). Ninety-five percent confidence intervals were estimated using bootstrap resampling. Model interpretation was performed using SHAP, including global feature rankings and representative local explanations (50).

### 2.10 Subgroup, sensitivity, and ablation analyses

Subgroup analyses evaluated performance by age group, sex, primary neurological diagnosis, mechanical ventilation status, and GCS category. Event counts were reported for each diagnostic group, and analyses with few deaths were designated exploratory. Sensitivity analyses examined alternative missingness thresholds, alternative feature-selection settings, exclusion of patients with extreme lengths of stay, and performance within major diagnostic groups. Ablation analyses compared the full model with reduced models based on demographics and comorbidities, vital signs and laboratory values, neurological variables, and combined neurological-systemic variables. Because SOFA is correlated with several constituent predictors, SHAP values for SOFA and its components were interpreted as dependent model attributions rather than independent effects.

### 2.11 Reproducibility and statistical software

Random seeds were fixed for data splitting, training, tuning, and bootstrap resampling. Preprocessing parameters, selected features, model configurations, and evaluation outputs were saved. Analyses were conducted using standard Python and R scientific-computing libraries. The MIMIC-IV development split contained approximately 811 deaths for 27 retained predictors (about 30 events per predictor), the internal test set contained 203 deaths, and eICU-CRD contained 686 deaths. A *post hoc* power calculation was not performed because uncertainty was quantified using event counts and bootstrap confidence intervals rather than a retrospective significance-based power estimate. Identifiable repository links and author metadata will be withheld during double-blind review and provided when permitted by the journal.

## 3 Results

### 3.1 Cohort selection and study population

After applying the inclusion and exclusion criteria, **6,842** eligible neurocritical-care patients were included from MIMIC-IV. Among them, **1,014** died during hospitalization, giving an event rate of **14.8%**. The external validation cohort included **5,236** eICU-CRD patients, of whom **686** died during hospitalization, giving an event rate of **13.1%**. The MIMIC-IV cohort was divided into an 8:2 stratified training-test split, with **5,474** patients and approximately **811** deaths in the development set and **1,368** patients and **203** deaths in the internal test set.

The cohort selection procedure is shown in Figure 1. The included population covered several neurocritical-care diagnoses. In MIMIC-IV, diagnostic-group death counts ranged from **41** for central nervous system infection to **266** for intracerebral hemorrhage; in eICU-CRD, they ranged from **26** to **177**, respectively (Supplementary Table S2). Results for the smallest diagnostic groups were therefore treated as exploratory. Compared with survivors, non-survivors were older and had lower GCS scores, higher SOFA scores, more frequent invasive mechanical ventilation and vasopressor use, higher lactate, worse renal-function markers, and more abnormal inflammatory and coagulation profiles (Table 1).

**Table 1:** Baseline characteristics of the study cohorts.

Variable	MIMIC-IV survivors *N* = 5,828	MIMIC-IV non-survivors *N* = 1,014	eICU-CRD survivors *N* = 4,550	eICU-CRD non-survivors *N* = 686
Age, years	63.4 ± 15.8	71.2 ± 13.9	64.1 ± 16.2	72.5 ± 13.4
Male sex, *n* (%)	3,146 (54.0)	574 (56.6)	2,485 (54.6)	397 (57.9)
Ischemic stroke, *n* (%)	1,684 (28.9)	241 (23.8)	1,376 (30.2)	171 (24.9)
Intracerebral hemorrhage, *n* (%)	1,112 (19.1)	266 (26.2)	842 (18.5)	177 (25.8)
Subarachnoid hemorrhage, *n* (%)	648 (11.1)	119 (11.7)	516 (11.3)	82 (12.0)
Traumatic brain injury, *n* (%)	1,032 (17.7)	206 (20.3)	781 (17.2)	139 (20.3)
Status epilepticus, *n* (%)	713 (12.2)	74 (7.3)	591 (13.0)	54 (7.9)
CNS infection, *n* (%)	286 (4.9)	41 (4.0)	214 (4.7)	26 (3.8)
Other neurological disorders, *n* (%)	353 (6.1)	67 (6.6)	230 (5.1)	37 (5.4)
GCS total score	11.8 ± 3.4	7.2 ± 3.6	11.5 ± 3.5	7.0 ± 3.7
SOFA score	5.6 ± 3.1	9.4 ± 4.0	5.9 ± 3.3	9.8 ± 4.2
Heart rate, beats/min	86.7 ± 18.5	98.4 ± 22.1	87.9 ± 18.8	99.1 ± 22.9
Mean arterial pressure, mmHg	82.6 ± 15.2	74.3 ± 17.8	83.1 ± 15.6	73.8 ± 18.1
Respiratory rate, breaths/min	19.7 ± 5.6	23.1 ± 7.0	20.1 ± 5.9	23.5 ± 7.3
SpO_2_, %	96.7 ± 3.1	94.2 ± 6.2	96.4 ± 3.4	93.8 ± 6.5
Creatinine, mg/dL	1.05 [0.78–1.42]	1.61 [1.04–2.48]	1.08 [0.81–1.47]	1.69 [1.10–2.62]
Blood urea nitrogen, mg/dL	22 [15–34]	38 [24–56]	24 [16–36]	41 [27–60]
Lactate, mmol/L	1.7 [1.1–2.5]	3.0 [1.9–5.1]	1.8 [1.2–2.7]	3.2 [2.0–5.4]
White blood cell count, 10^9^/L	10.8 ± 4.9	14.2 ± 6.7	11.1 ± 5.1	14.8 ± 7.0
Platelet count, 10^9^/L	214 ± 91	176 ± 84	219 ± 94	171 ± 86
Invasive mechanical ventilation, *n* (%)	2,176 (37.3)	721 (71.1)	1,634 (35.9)	505 (73.6)
Vasopressor use, *n* (%)	1,241 (21.3)	548 (54.0)	1,038 (22.8)	391 (57.0)
Renal replacement therapy, *n* (%)	214 (3.7)	114 (11.2)	189 (4.2)	83 (12.1)

Values are presented as mean ± standard deviation, median [interquartile range], or *n* (%). CNS, central nervous system; GCS, Glasgow Coma Scale; SOFA, Sequential Organ Failure Assessment; SpO_2_, peripheral oxygen saturation.

### 3.2 Feature selection and retained predictors

After cross-database harmonization, **64** candidate variables were retained from the fixed 24-h interface. Variables exceeding the missingness threshold were removed before modeling. LASSO regression selected **27** predictors in the development cohort (Table 2).

**Table 2:** Candidate variables and selected feature domains.

Feature domain	Candidate variables	Selected variables	Representative selected predictors
Demographics	5	3	Age, sex, admission type
Primary neurological diagnosis	7	5	Intracerebral hemorrhage, traumatic brain injury, ischemic stroke
Comorbidities	12	4	Chronic kidney disease, heart failure, malignancy, diabetes
Vital signs	13	6	Mean arterial pressure, heart rate, respiratory rate, SpO_2_, temperature, systolic blood pressure
Laboratory indices	19	7	Lactate, creatinine, blood urea nitrogen, white blood cell count, platelet count, sodium, international normalized ratio
Neurological status	4	2	GCS total score, GCS motor component
Early ICU therapies	4	3	Mechanical ventilation, vasopressor use, renal replacement therapy
Total	64	27	–

GCS, Glasgow Coma Scale; ICU, intensive care unit; SpO_2_, peripheral oxygen saturation.

The selected predictors covered neurological status, respiratory support, circulation, renal function, inflammation, coagulation, and early treatment intensity. Frequently retained variables included age, total GCS score, GCS motor component, SOFA score, mechanical ventilation, vasopressor use, mean arterial pressure, respiratory rate, oxygen saturation, lactate, creatinine, blood urea nitrogen, platelet count, white blood cell count, glucose, sodium, international normalized ratio, and primary neurological diagnosis.

### 3.3 Model performance in the internal test cohort

All candidate models showed useful discrimination in the internal MIMIC-IV test cohort (Table 3; Figure 3). Ensemble tree models performed better than logistic regression and multilayer perceptron. LightGBM achieved the best overall internal performance, with an AUROC of **0.841** and an AUPRC of **0.518**. CatBoost and XGBoost had similar performance.

**Table 3:** Internal test performance in MIMIC-IV.

Model	AUROC	AUPRC	Sensitivity	Specificity	PPV	NPV	F1 score	Brier score
Logistic regression	0.790	0.404	70.9%	72.4%	30.4%	93.5%	0.424	0.107
Random forest	0.817	0.466	73.5%	74.8%	34.1%	94.1%	0.466	0.098
XGBoost	0.833	0.503	76.5%	75.5%	36.2%	94.7%	0.490	0.093
LightGBM	**0.841**	**0.518**	**77.2%**	**76.0%**	**37.0%**	**94.9%**	**0.501**	**0.090**
CatBoost	0.836	0.512	76.8%	75.8%	36.6%	94.8%	0.497	0.092
Multilayer perceptron	0.804	0.437	71.8%	73.9%	32.5%	93.8%	0.448	0.103

AUROC, area under the receiver operating characteristic curve; AUPRC, area under the precision-recall curve; PPV, positive predictive value; NPV, negative predictive value. Bold values indicate the best performance among the compared models for each metric.

**Figure 3:**
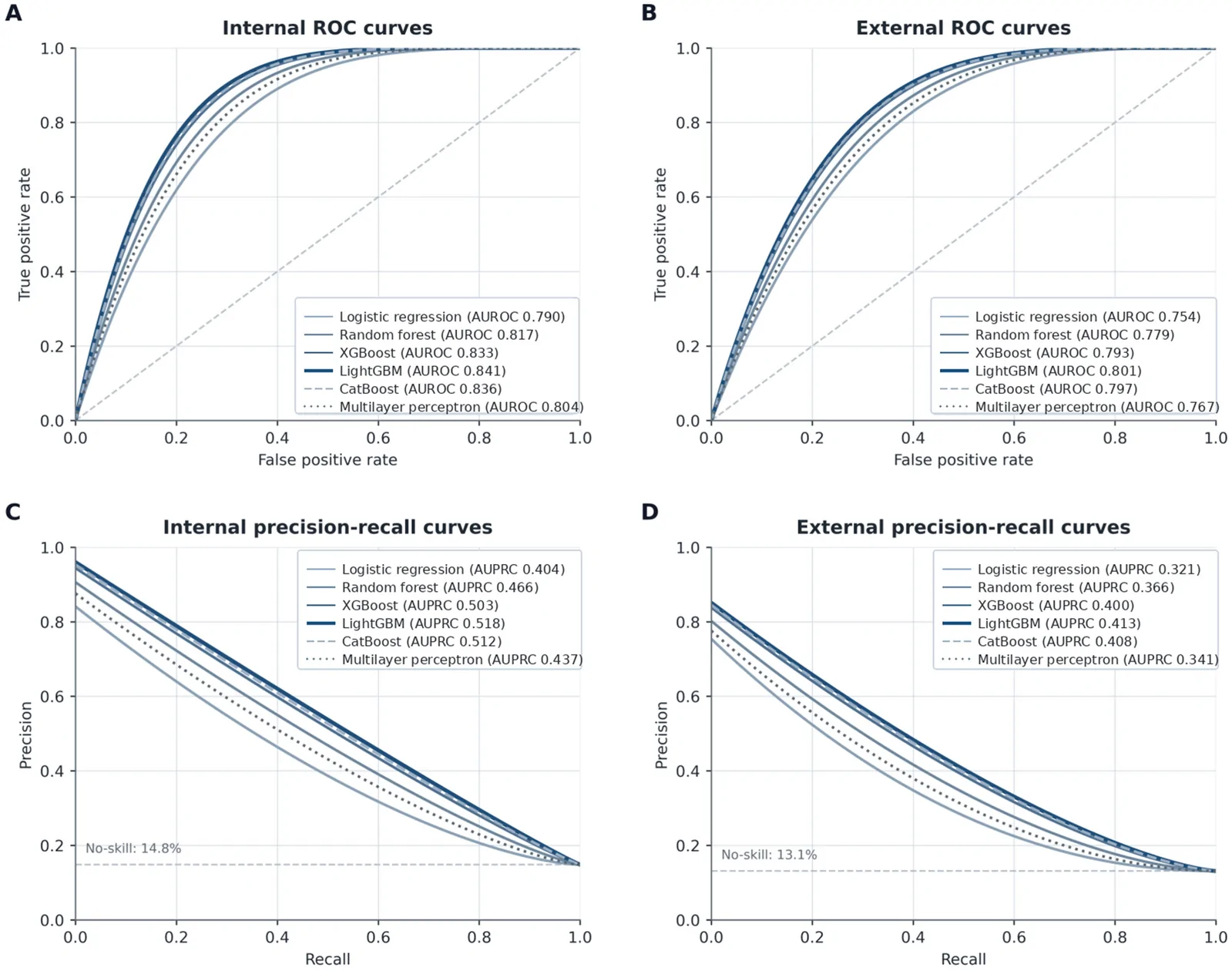
Discrimination performance of candidate models in the internal test cohort and external validation cohort. **(A, B)** Show receiver operating characteristic curves, and **(C, D)** show precision-recall curves. AUROC and AUPRC are reported for logistic regression, random forest, XGBoost, LightGBM, CatBoost, and multilayer perceptron models. LightGBM achieved the strongest overall discrimination and was selected as the final model after considering calibration, external transportability, clinical utility, and interpretability.

At the prespecified threshold, LightGBM achieved sensitivity of **77.2%**, specificity of **76.0%**, positive predictive value of **37.0%**, negative predictive value of **94.9%**, and F1 score of **0.501**. The high negative predictive value indicates that the model was better at identifying lower-risk patients than confirming death in a lower-prevalence outcome setting. Conversely, **63.0%** of patients classified as high risk in the internal test set survived to discharge.

### 3.4 External validation in eICU-CRD

In eICU-CRD, the LightGBM model retained moderate-to-good discrimination (Table 4; Figure 3). The external AUROC was **0.801**, and the AUPRC was **0.413**. The reduction from internal performance was expected given differences in hospital systems, coding, case mix, and measurement patterns between databases.

**Table 4:** External validation performance in eICU-CRD.

Model	AUROC	AUPRC	Sensitivity	Specificity	PPV	NPV	F1 score	Brier score
Logistic regression	0.754	0.321	66.8%	70.9%	26.3%	93.3%	0.377	0.114
Random forest	0.779	0.366	69.4%	72.5%	28.2%	94.0%	0.401	0.109
XGBoost	0.793	0.400	72.0%	73.3%	29.7%	94.5%	0.421	0.104
LightGBM	**0.801**	**0.413**	**72.8%**	**73.9%**	**30.3%**	**94.7%**	**0.428**	**0.101**
CatBoost	0.797	0.408	72.3%	73.5%	29.9%	94.6%	0.424	0.103
Multilayer perceptron	0.767	0.341	68.0%	72.1%	27.4%	93.6%	0.390	0.112

AUROC, area under the receiver operating characteristic curve; AUPRC, area under the precision-recall curve; PPV, positive predictive value; NPV, negative predictive value. Bold values indicate the best performance among the compared models for each metric.

At the same operating threshold, the external sensitivity was **72.8%**, specificity was **73.9%**, positive predictive value was **30.3%**, negative predictive value was **94.7%**, and F1 score was **0.428**. Accordingly, **69.7%** of externally flagged patients survived to discharge. The operating point therefore preserved risk-stratification ability but had a substantial false-positive burden and should not be used as a stand-alone trigger for prognostic or treatment-limitation decisions.

### 3.5 Calibration and clinical utility

Calibration results are summarized in Table 5 and Figure 4. In the internal test cohort, the LightGBM model had a Brier score of **0.090**, calibration intercept of **0.024**, and calibration slope of **0.91**. In the external validation cohort, the Brier score was **0.101**, calibration intercept was **0.050**, and calibration slope was **0.84**. The calibration curve showed mild overprediction in the highest-risk decile but acceptable agreement in low- and intermediate-risk ranges.

**Table 5:** Calibration and decision-curve summary for the final LightGBM model.

Cohort	AUROC	AUPRC	Brier score	Calibration intercept	Calibration slope	DCA positive net-benefit range
MIMIC-IV internal test	0.841	0.518	0.090	0.024	0.91	0.09–0.58
eICU-CRD external validation	0.801	0.413	0.101	0.050	0.84	0.11–0.51

DCA, decision curve analysis.

**Figure 4:**
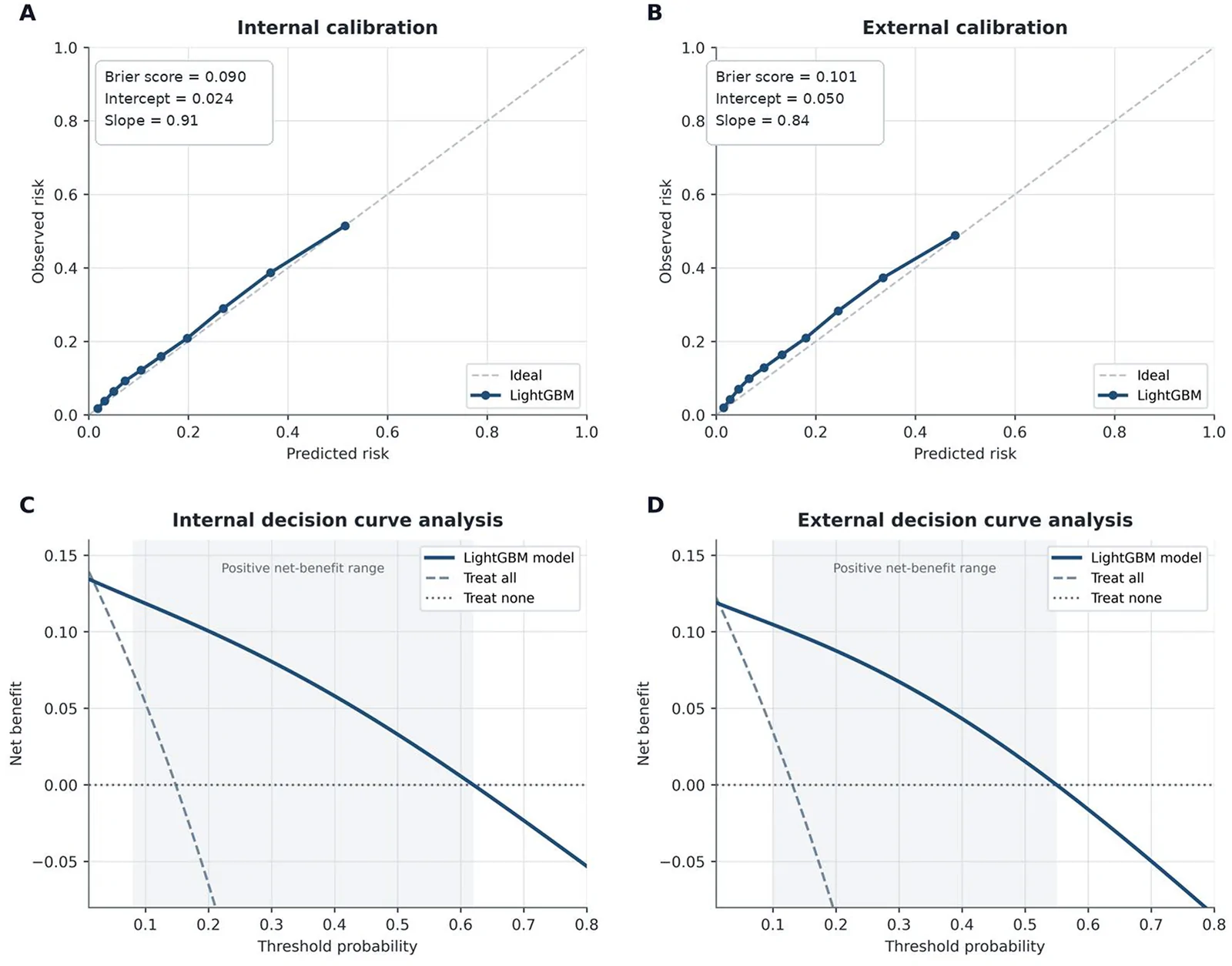
Calibration and decision-curve analysis of the final LightGBM model. **(A, B)** Compare predicted and observed in-hospital mortality risk in the internal test cohort and external validation cohort, respectively. **(C, D)** Show decision-curve analysis across threshold probabilities, using treat-all and treat-none strategies as reference lines. The shaded regions indicate threshold ranges in which model-guided risk stratification yielded positive net benefit.

DCA showed positive net benefit for LightGBM across the evaluated threshold range (Figure 4). In the internal test cohort, the model outperformed the treat-all and treat-none strategies across thresholds of approximately **0.09–0.58**; in external validation, positive net benefit was preserved across thresholds of approximately **0.11–0.51**. These exploratory curves do not establish clinical benefit because the consequences of acting on a positive prediction were not tested prospectively.

### 3.6 Model interpretation

SHAP analysis identified total GCS score, invasive mechanical ventilation, age, lactate, SOFA score, vasopressor use, creatinine, respiratory rate, mean arterial pressure, and oxygen saturation as the most influential predictors (Figure 5). Lower GCS score, mechanical ventilation, elevated lactate, vasopressor exposure, renal dysfunction, hypotension, tachypnea, and lower oxygen saturation were associated with higher predicted mortality risk. The dependence plots further suggested monotonic or near-monotonic risk gradients for several clinically interpretable variables, including GCS total score, lactate, and mean arterial pressure.

**Figure 5:**
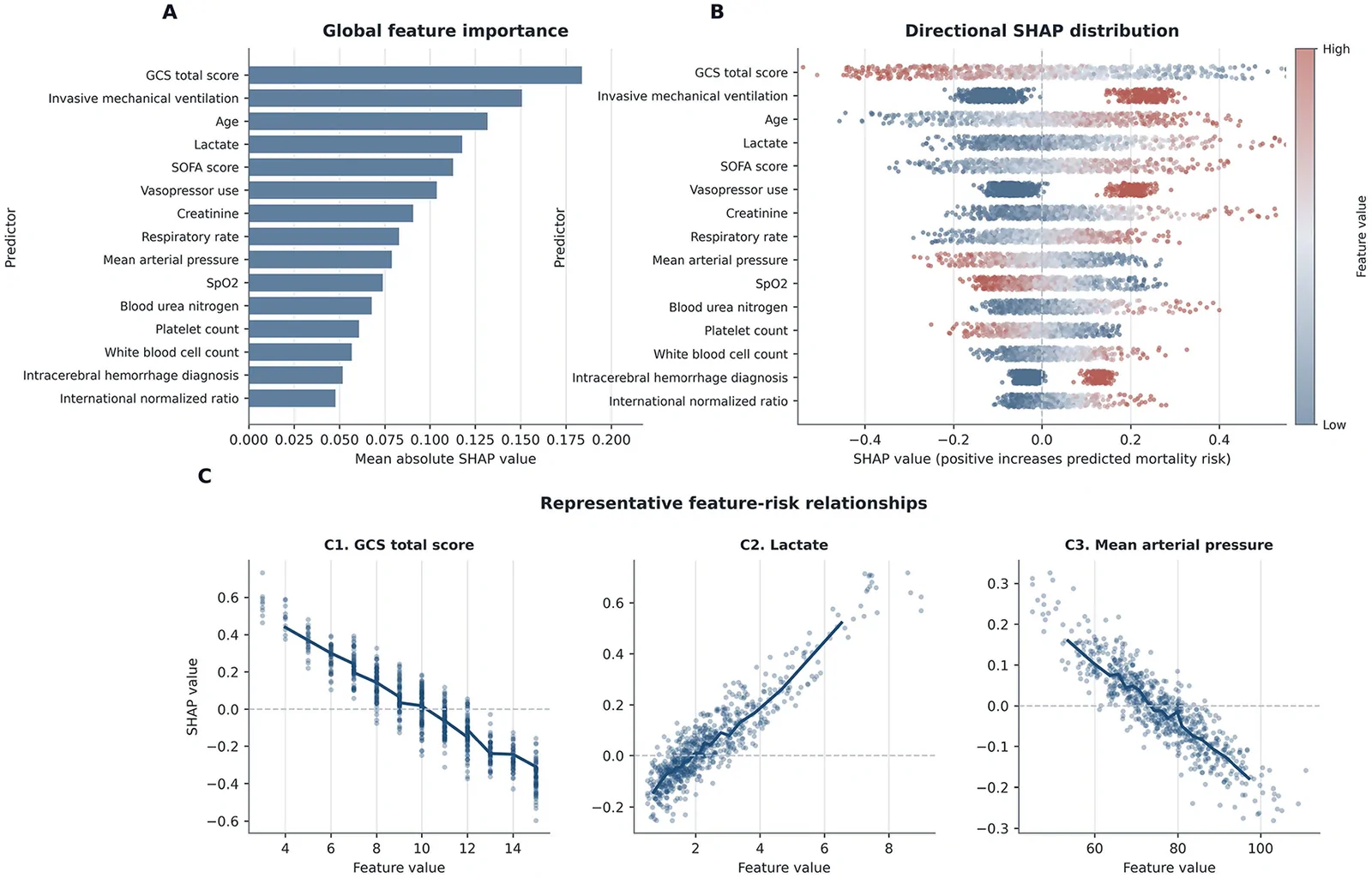
SHAP-based interpretation of the final LightGBM model. **(A)** Ranks predictors by mean absolute SHAP value. **(B)** Shows the directional SHAP distribution, where positive SHAP values increase predicted mortality risk and color indicates the original feature value. **(C)** Presents representative feature-risk relationships for GCS total score, lactate, and mean arterial pressure. Because SOFA and several component variables are correlated, their SHAP values describe dependent model attributions and should not be interpreted as independent or causal effects.

The SHAP profile was clinically coherent. Neurological impairment was a dominant signal, but respiratory compromise, circulatory instability, renal dysfunction, and systemic stress also contributed. Because SOFA overlaps with GCS, creatinine, vasopressor use, mechanical ventilation, and other constituent variables, their SHAP values are not independent and should not be summed or interpreted as separate causal contributions. Local explanations showed that patients could reach similar predicted risks through different predictive pathways.

### 3.7 Subgroup analyses

The final model maintained AUROC values generally above **0.76** across age, sex, primary diagnosis, mechanical ventilation status, and GCS-defined strata. Performance was strongest in intracerebral hemorrhage and traumatic brain injury and lower in smaller or more heterogeneous groups, including status epilepticus and central nervous system infection. Because these groups contained only 74 and 41 deaths in MIMIC-IV and 54 and 26 deaths in eICU-CRD, respectively, subgroup estimates were considered exploratory rather than definitive.

The model also remained informative among mechanically ventilated patients and among patients with low GCS scores. This finding suggests that systemic physiological variables added prognostic information even when neurological severity was already high.

### 3.8 Sensitivity and ablation analyses

Sensitivity analyses showed limited variation under alternative preprocessing and feature-selection settings. Changing the missingness threshold produced external AUROC values ranging from **0.789** to **0.807**. Using all retained harmonized variables slightly improved internal performance but did not improve external validation.

Ablation analyses showed that neurological variables alone were not sufficient. A neurological-only model achieved an external AUROC of **0.716**. A model using vital signs and laboratory indices achieved an AUROC of **0.760**. Combining neurological and systemic variables improved AUROC to **0.792**, while the full model reached **0.801**. These results support the value of integrating brain-specific and extracranial physiological information.

## 4 Discussion

### 4.1 Principal findings

In this retrospective multicenter study, we developed and externally validated an interpretable ML model for prediction of in-hospital mortality at a fixed 24-h ICU landmark. Using only variables available within the first 24 h after ICU admission, the LightGBM model achieved an AUROC of **0.841** in the internal test cohort and **0.801** in the independent external validation cohort. Calibration and SHAP explanations were acceptable, but the external PPV of **30.3%** indicates that most patients classified as high risk at the reported operating point survived to discharge. The model should therefore be viewed as a risk-stratification instrument rather than a definitive prognostic test.

Three findings are important. First, the model maintained performance after transfer from MIMIC-IV to eICU-CRD, despite differences in coding, measurement patterns, and institutional practice. Second, the leading predictors were clinically interpretable and included neurological severity, respiratory support, systemic organ dysfunction, and metabolic stress. Third, ablation analysis showed that neurological variables alone were inferior to models that integrated neurological and systemic information. These findings support the view that early mortality risk in neurocritical care is multidimensional.

### 4.2 Clinical interpretation of the 24-h prediction window

The fixed 24-h prediction interface was chosen for clinical and methodological reasons. Clinically, the first ICU day is when physicians assess severity, decide monitoring intensity, initiate organ support, and discuss prognosis. A prediction model at this time point may therefore support decisions before the subsequent hospital course is fully known.

Methodologically, a fixed early window reduces information leakage. In retrospective ICU studies, variables recorded after deterioration or treatment escalation can make a model appear accurate while limiting its usefulness at the intended decision point. By using only first-day variables, the present model more closely reflects information available to clinicians when early risk stratification is needed.

The 24-h window also balances timeliness and information richness. Admission-only models may miss early laboratory evolution and initial treatment response. Later models may be less useful for early intervention. First-day data capture neurological status, respiratory function, hemodynamics, renal function, inflammatory burden, and treatment intensity in a clinically interpretable form.

### 4.3 Comparison with previous work

Previous ML studies in neurocritical care have often focused on single disease groups, including stroke, traumatic brain injury, and subarachnoid hemorrhage (14–18). These studies show that ML can extract prognostic information from ICU data and that model interpretation can identify clinically recognizable risk factors. The present study extends this line of work to a broader neurocritical-care population, which better reflects mixed ICU practice.

Compared with general ICU mortality models, this study emphasizes neurological context while still accounting for extracranial organ dysfunction (19–21, 24–26). Related machine-learning studies in extracorporeal membrane oxygenation populations have likewise identified lactate and other physiologic measurements as important predictive features, although their populations and outcomes differ substantially from those studied here (51, 52). These comparisons support the value of multisystem physiology while underscoring that performance and feature rankings are context dependent.

### 4.4 Interpretation of model predictors

GCS score was the most influential predictor, as expected. Impaired consciousness is a direct marker of neurological severity and may also reflect airway risk, brainstem dysfunction, seizure activity, sedation, or metabolic disturbance. However, the model did not depend on GCS alone. Mechanical ventilation and vasopressor use are markers of both illness severity and clinician treatment decisions, and they may vary according to local practice. SOFA is also a composite of several variables represented separately in the model. Consequently, SHAP values for SOFA and its components describe the fitted model's allocation of risk but do not represent independent or causal effects.

Renal markers, including creatinine and blood urea nitrogen, also contributed to predicted mortality. Kidney dysfunction may reflect hypoperfusion, nephrotoxic exposure, inflammation, or broader multi-organ failure. Lactate represented metabolic stress and tissue hypoperfusion. White blood cell count and platelet count captured inflammatory and coagulation-related abnormalities. Together, these predictors suggest that mortality risk is shaped by both primary neurological injury and systemic physiological reserve.

### 4.5 Calibration and clinical utility

Discrimination alone is insufficient for clinical use. A model may rank patients well but still overestimate or underestimate absolute risk. The calibration analysis showed acceptable agreement between predicted and observed risk, although mild overprediction was present in the highest-risk range. This point should be considered if the model is used for absolute risk communication.

DCA suggested potential net benefit over treat-all and treat-none strategies across the evaluated thresholds, but the analysis does not account for the specific harms of false-positive mortality predictions. At the external operating point, nearly 70% of patients classified as high risk survived to discharge. Such predictions could contribute to premature prognostic pessimism if used without clinical safeguards. The threshold is therefore descriptive and should not be used to trigger treatment limitation or WLST. Prospective impact studies are required to define an action, quantify alarm burden, and determine whether model-assisted care improves outcomes.

### 4.6 Subgroup and ablation findings

Subgroup results showed that the model remained useful across age, sex, diagnostic, ventilation, and GCS strata. Performance was lower in smaller or more heterogeneous diagnostic groups, which is expected when event counts and disease mechanisms vary. These findings support further validation in larger neurocritical-care cohorts.

Ablation analysis provided an important clinical message. Neurological status alone did not capture the full mortality signal. Adding systemic physiology improved external validation performance, and the full model performed best. This supports the assumption that early mortality risk in neurocritical care is partly driven by extracranial organ dysfunction and treatment intensity.

### 4.7 Implications for clinical decision support

The model is not intended to replace clinical judgment. Its potential role is limited to supporting structured reassessment and identifying patients who may warrant closer observation. A high predicted risk must not be interpreted as futility and must not be used in isolation to justify treatment limitation, do-not-resuscitate orders, or WLST. Any implementation should include safeguards against automation bias and require prospective evaluation of false-positive consequences.

Interpretability is important for this role. A probability score is difficult to use if clinicians cannot understand its basis. SHAP explanations can show whether a high predicted risk is mainly driven by depressed consciousness, respiratory failure, shock, renal dysfunction, inflammatory burden, or a combination of these factors. This may help clinicians judge whether a prediction is plausible and whether any component is modifiable.

### 4.8 Limitations

This study has limitations. First, it was retrospective and used routinely collected EHR data. Residual confounding, measurement bias, and coding errors cannot be excluded. Neurocritical-care diagnoses were identified from ICD codes without independent adjudication; variation in code completeness, principal-diagnosis assignment, and billing practice may therefore have introduced selection and misclassification bias.

Second, observed in-hospital mortality could not be separated into deaths following WLST or comfort-care transition and deaths despite continued life-sustaining therapy. Because GCS, age, neurological severity, ventilation, and vasopressor use can influence both prognosis and treatment-limitation decisions, the model may partly reproduce historical WLST practice and self-fulfilling prophecy bias. It must not be interpreted as a model of untreated biological mortality.

Third, variable harmonization across MIMIC-IV and eICU-CRD required mapping between different database structures, and local thresholds for intubation, vasopressor initiation, laboratory testing, and ICU admission may differ. Detailed variable-level missingness estimates are not available for every candidate predictor in the present report, which limits reproducibility. Although imputation was confined to training data and sensitivity analyses varied missingness thresholds, a complete-case analysis was not undertaken because measurement availability is clinically informative and complete-case restriction may introduce selection bias.

Fourth, SOFA overlaps with several constituent predictors, so SHAP attributions for correlated variables are not independent. Fifth, the model used a fixed 24-h interface and did not update predictions during the later ICU course. Sixth, in-hospital mortality does not capture long-term neurological, cognitive, psychiatric, or functional outcomes; prior evidence shows that substantial disability may persist among survivors (53). Finally, both databases represent United States practice. Prospective evaluation is required in other countries and health systems, including Latin America, before claims of international generalizability or clinical impact can be made.

### 4.9 Conclusions

An interpretable ML model using routinely available first-day ICU data stratified the risk of in-hospital mortality in neurocritical-care patients and retained discrimination in external validation. The model should not be used as a stand-alone basis for prognostic pessimism, treatment limitation, or WLST, particularly given its low positive predictive value and the unresolved influence of care-limitation practices. Prospective validation should include patient-centered neurological and functional outcomes and should be conducted across diverse countries and health systems, including Latin America.

## Data Availability

Publicly available datasets were analyzed in this study. This data can be found here: The datasets used in this study are the Medical Information Mart for Intensive Care IV (MIMIC-IV) and the eICU Collaborative Research Database (eICU-CRD). Both datasets are available to credentialed researchers through the PhysioNet repository under their respective data-use agreements. The authors are not permitted to redistribute the underlying patient-level records.
