# Supplementary material for: Development and external validation of an interpretable machine learning model for early prediction of in-hospital mortality in neurocritical care
Source: Front Neurol. 2026 Sep 24;17:1888888. doi: 10.3389/fneur.2026.1888888 (PMC13646724; doi:10.3389/fneur.2026.1888888)
Supplement: Supplementary file 1 [file Supplementary_file_1.pdf]

# Supplementary Material

*Development and external validation of an interpretable machine learning model for early prediction of in-hospital mortality in neurocritical care*

**Xiao Cheng, Jianxin Ye, and Hongen Wei**

This file contains the supplementary reporting checklist and diagnostic-group sample-size summary cited in the main article.

## Supplementary Table S1. TRIPOD reporting checklist

| Item | Topic | Location in revised manuscript |
|---|---|---|
| 1 | Title | Title identifies model development and external validation. |
| 2 | Abstract | Structured abstract reports design, data sources, participants, predictors, outcome, performance, and conclusions. |
| 3 | Background and objectives | Introduction. |
| 4 | Source of data | Materials and Methods: Data sources. |
| 5 | Participants | Materials and Methods: Study population. |
| 6 | Outcome | Materials and Methods: Outcome definition. |
| 7 | Predictors | Materials and Methods: Fixed 24-hour prediction interface. |
| 8 | Sample size | Materials and Methods: Reproducibility and statistical software; Results: Cohort selection. |
| 9 | Missing data | Materials and Methods: Data preprocessing and feature harmonization; Limitations. |
| 10 | Statistical analysis methods | Materials and Methods: Model development and performance evaluation. |
| 11 | Risk groups / operating point | Materials and Methods: Model development and performance evaluation. |
| 12 | Development versus validation | Materials and Methods: Internal and external validation. |
| 13 | Participant flow | Results: Cohort selection; Figure 1. |
| 14 | Participant characteristics | Table 1. |
| 15 | Model development | Results: Feature selection and retained predictors. |
| 16 | Model specification | Materials and Methods: Model development; Table 2. |
| 17 | Model performance | Results; Tables 3–5; Figures 3–4. |
| 18 | Model updating | No updating was performed in eICU-CRD. |
| 19 | Limitations | Discussion: Limitations. |
| 20 | Interpretation | Discussion: Principal findings and clinical implications. |
| 21 | Implications | Discussion: Implications for clinical decision support and Conclusions. |
| 22 | Supplementary information | Reproducibility statement and Appendices. |

## Supplementary Table S2. Diagnostic-group sample sizes and mortality events

| Diagnostic group | MIMIC-IV, N | MIMIC-IV deaths | eICU-CRD, N | eICU-CRD deaths |
|---|---|---|---|---|
| Ischemic stroke | 1,925 | 241 | 1,547 | 171 |
| Intracerebral hemorrhage | 1,378 | 266 | 1,019 | 177 |
| Subarachnoid hemorrhage | 767 | 119 | 598 | 82 |
| Traumatic brain injury | 1,238 | 206 | 920 | 139 |
| Status epilepticus | 787 | 74 | 645 | 54 |
| Central nervous system infection | 327 | 41 | 240 | 26 |
| Other neurological disorders | 420 | 67 | 267 | 37 |
| Total | 6,842 | 1,014 | 5,236 | 686 |

Abbreviations: MIMIC-IV, Medical Information Mart for Intensive Care IV; eICU-CRD, eICU Collaborative Research Database. Diagnostic groups were defined by the prespecified ICD-based cohort rules described in the main text.
